# A method to generate capture baits for targeted sequencing

**DOI:** 10.1093/nar/gkad460

**Published:** 2023-06-01

**Authors:** Balaji Sundararaman, Alisa O Vershinina, Samantha Hershauer, Joshua D Kapp, Shelby Dunn, Beth Shapiro, Richard E Green

**Affiliations:** Department of Biomolecular Engineering, University of California Santa Cruz, Santa Cruz, CA 95064, USA; Howard Hughes Medical Institute, University of California Santa Cruz, Santa Cruz, CA 95064, USA; Department of Ecology and Evolutionary Biology, University of California Santa Cruz, Santa Cruz, CA 95064, USA; Department of Biomolecular Engineering, University of California Santa Cruz, Santa Cruz, CA 95064, USA; Howard Hughes Medical Institute, University of California Santa Cruz, Santa Cruz, CA 95064, USA; Department of Ecology and Evolutionary Biology, University of California Santa Cruz, Santa Cruz, CA 95064, USA; Department of Ecology and Evolutionary Biology, University of California Santa Cruz, Santa Cruz, CA 95064, USA; Department of Biomolecular Engineering, University of California Santa Cruz, Santa Cruz, CA 95064, USA; Department of Ecology and Evolutionary Biology, University of California Santa Cruz, Santa Cruz, CA 95064, USA; Howard Hughes Medical Institute, University of California Santa Cruz, Santa Cruz, CA 95064, USA; UCSC Genomics Institute, University of California Santa Cruz, Santa Cruz, CA 95064, USA; Department of Biomolecular Engineering, University of California Santa Cruz, Santa Cruz, CA 95064, USA; UCSC Genomics Institute, University of California Santa Cruz, Santa Cruz, CA 95064, USA

## Abstract

Hybridization capture approaches allow targeted high-throughput sequencing analysis at reduced costs compared to shotgun sequencing. Hybridization capture is particularly useful in analyses of genomic data from ancient, environmental, and forensic samples, where target content is low, DNA is fragmented and multiplex PCR or other targeted approaches often fail. Here, we describe a DNA bait synthesis approach for hybridization capture that we call *C*ircular *N*ucleic acid *E*nrichment *R*eagent, or CNER (pronounced ‘*snare’*). The CNER method uses rolling-circle amplification followed by restriction digestion to discretize microgram quantities of hybridization probes. We demonstrate the utility of the CNER method by generating probes for a panel of 23 771 known sites of single nucleotide polymorphism in the horse genome. Using these probes, we capture and sequence from a panel of ten ancient horse DNA libraries, comparing CNER capture efficiency to a commercially available approach. With about one million read pairs per sample, CNERs captured more targets (90.5% versus 66.5%) at greater mean depth than an alternative commercial approach.

## Graphical Abstract

**Graphical Abstract:**
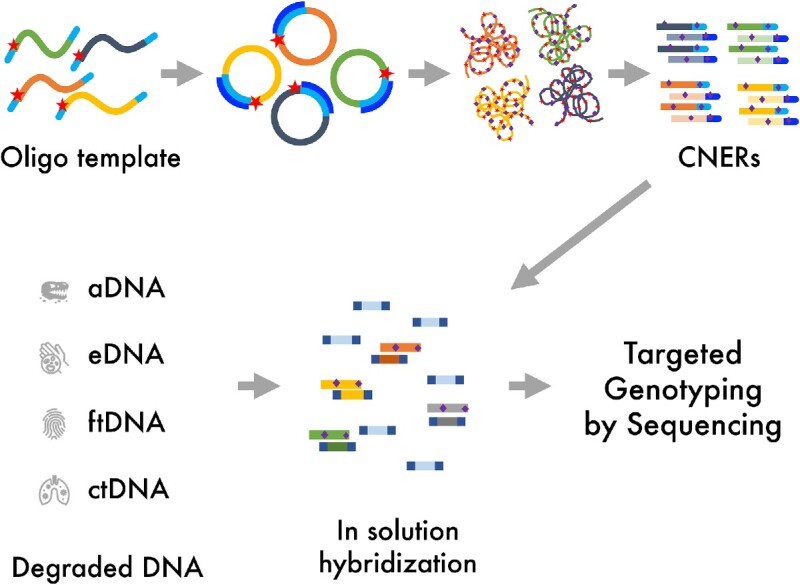

## INTRODUCTION

Compared with whole-genome sequencing, targeted sequencing is a cost-effective method for analyzing specific genomic regions (1). Targeted sequencing has wide application in diagnostics, metagenomic, phylogenetic, ancient and environmental DNA studies, and forensics (2,3). In targeted sequencing, regions of interest are enriched by hybridization capture using target-specific probes or by PCR amplification using target-specific primers, followed by high-throughput next-generation sequencing (NGS). Hybridization capture methods overcome drawbacks of PCR-based target enrichment, including scalability to a large number of targets, PCR failure and PCR artifacts (1,2).

Pioneering hybridization capture experiments used DNA arrays to enrich for targeted sequencing of human samples (4–7) and Neanderthal ancient DNA (aDNA) (8). In these array-based hybridization capture methods, NGS library molecules were hybridized to a microarray imprinted with probes targeting human exons. After washing non-hybridized library molecules off the surface of the array, captured molecules were eluted and sequenced (4–8). Array-based hybridization capture expanded the capability to millions of target regions, beyond what is achievable with PCR-based enrichment methods (1–3). However, array-based capture is labor and time-intensive and requires large amounts of input DNA as well as specialized instrumentation for capture.

In-solution hybridization capture is currently the most commonly used method of targeted sequencing due to the commercial availability of capture probes and the simplicity of the approach (2,3). In-solution hybridization capture uses biotinylated DNA or RNA molecules (baits) to capture target regions (1–3,9). A molar excess of biotinylated baits is hybridized with NGS libraries in solution. The resulting library-bait heteroduplexes are captured on streptavidin-coated magnetic beads. Unbound non-target molecules are washed away, and target molecules are recovered for sequencing (9,10).

Current bait synthesis methods require large-scale oligonucleotide chemical synthesis and/or *in vitro* transcription. Both RNA and DNA bait generation requires synthesizing template oligonucleotides using phosphoramidite chemistry. Microarray-based synthesis generates oligonucleotides in femtomole scales with chemical coupling error rates of 10^−2^–10^−3^ (11,12). Templates synthesized at small-scale require enzymatic amplification before use in hybridization capture. For RNA baits, PCR amplified oligo templates are transcribed *in vitro* into biotinylated RNA baits as initially described by Gnrike *et al.* (9). However, *in vitro* transcription using T7 RNA polymerase can lead to amplification biases based on the templates’ sequence, length, and GC content (13,14). For DNA baits, either a small-scale template pool is enzymatically amplified (Twist Biosciences product sheet) or each bait is individually manufactured at scale (IDT product sheet).

**Figure 1.**
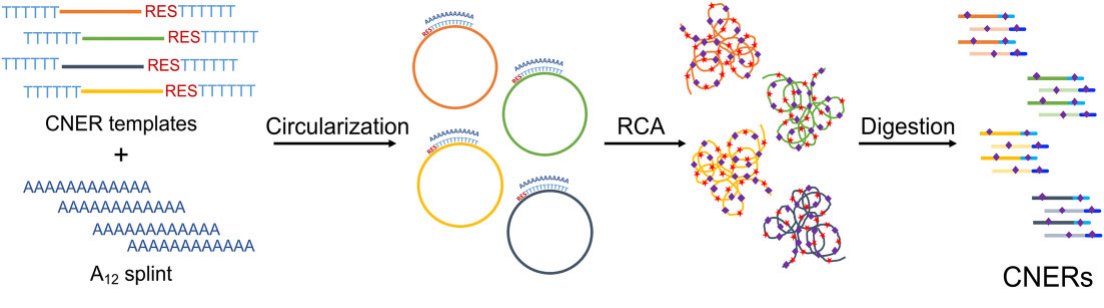
*C*ircular *N*ucleic acid *E*nrichment *R*eagent method. An oligonucleotide template pool containing restriction enzyme recognition sites (RES) and oligo-dT linkers is circularized by an oligo-dA splint adapter mediated ligation. Circularized templates are isothermally amplified using oligo-dA and oligo-dT oligos by rolling circle amplification (RCA). RCA products are then digested with restriction enzymes to generate CNERs. CNERs generate both strands (dark and light shades of colors) of the templates. Biotinylated nucleotides (purple diamonds) are incorporated during amplification.

We present a cost-effective, large-scale DNA bait synthesis method that we call *C*ircular *N*ucleic acid *E*nrichment *R*eagent, or CNER (pronounced as *snare*). The CNER method involves circularization of target template oligos that contain a linker region to promote circularization via splint-ligation and a rare-cutter restriction enzyme site for subsequent discretization of the capture probes. Circularized templates are isothermally amplified by rolling circle amplification (RCA) with the inclusion of biotinylated nucleotides. The long RCA products are discretized into single biotinylated baits by restriction digestion (Figure 1). The resulting biotinylated CNER probes can be generated in microgram quantities and used for capture enrichments on streptavidin-coated beads.

Here, we demonstrate the use of the CNER method for targeted genotyping by producing a set of CNER probes to capture 23771 SNPs in the horse genome. We use these CNERs to capture target SNPs from ten ancient horse DNA libraries of varying endogenous DNA content and DNA degradation levels. We show that the CNERs effectively perform target enrichment even in highly degraded ancient samples comparably to or better than commercially made baits and at a fraction of the cost.

## MATERIALS AND METHODS

### DNA isolation

We selected ten ancient horse samples of varying DNA preservation (details in Supplementary Table S1 and in (15)) to test the performance of the CNER method. The samples date to the Late Pleistocene between 10 000 and 50 000 years ago, based on stratigraphic information and directly radiocarbon dated collagen (Supplementary Table S1 and in (15)). We extracted ancient DNA following (16) in a dedicated ancient DNA laboratory at the UC Santa Cruz Paleogenomics Laboratory (PGL) and following standard protocols for handling ancient DNA (17).

We isolated DNA from four modern domestic horses for capture optimization using blood samples drawn in May/June 2017 during routine veterinary checks. We used the DNeasy Blood & Tissue kit (Qiagen) following the manufacturer's protocol.

### Sequencing library preparation

We prepared NGS libraries from each horse extract using the Santa Cruz Reaction (SCR) (18). For the modern horse, we fragmented genomic DNA using 0.02U DNase I (Thermo Fisher) at 15°C for 15 min with MgCl_2_ before proceeding with the SCR. We prepared ancient horse DNA libraries in the dedicated clean at the PGL. For both ancient and modern samples, we divided adapter-ligated DNA into three aliquots before PCR amplification. We PCR-amplified ancient DNA libraries with Illumina unique dual index primers (19) using 2x AmpliTaq Gold 360 master mix (Thermo Fisher) at 95°C for 10 min, followed by 10–15 cycles of 95°C for 30 s, 60°C for 30 s, 72°C for 1 min, with a final extension at 72°C for 7 min followed by a hold at 12°C. We PCR amplified the modern horse libraries with Illumina unique dual index primers using 2× KAPA HiFi master mix (Roche) at 98°C for 3 min, followed by 13 cycles of 98°C for 30 s, 65°C for 20 s, 72°C for 20 s, with a final extension at 72°C for 3 min then hold at 12°C. We purified the amplified libraries with SPRI (20) beads at 0.8× ratio for the modern horse and at 1.2x for the ancient horses, quantified the DNA using Qubit 1× HS assay (Thermo Fisher), and determined library size by Fragment Analyzer (Agilent).

### Horse SNP panel design

We designed the horse SNP panel for target enrichment of known nuclear SNPs based on the SNP ascertainment scheme described in (15). Briefly, we genotyped Batagai (21), CGG10022 (22), YG188.42/YT03-40 and YG303.325 (both from (15)) ancient horse genomes mapped to EquCab2 (GenBank: GCA_000002305.1; (23)) as described in (15), using samtools v.1.7 utilities mpileup and bcftools (24), AntCaller v1.1 (25), and GATK HaplotypeCaller 3.7 (26). We intersected variant calls from all three programs using VCFtools v0.1.16 vcf-isec (27). In downstream analyses, we used only variants called by all three programs. We also removed variants with <20 base call quality, <5X read coverage, location within 5 bp of indels, singletons and homozygous alternative alleles in all four ancient horse genomes. We selected SNPs located outside of gene boundaries and repetitive regions using the filtering strategy described in (15).

We selected the candidate set of 26944 variant loci for bait designing by Arbor Biosciences. Arbor provided us a list of 74 385 candidate baits. We filtered these to limit to 60K baits based on the chosen synthesis tier. We chose baits with 20–80% GC content, filtered out baits containing repeats using RepeatMasker and baits with strong secondary structures (Δ*G* > –9 kcal/mol). After filtering, we chose a final list of baits to target 22 619 variant loci to proceed with Arbor myBaits generation. The final Arbor panel targeted 2583 SNPs using one bait, 3391 SNPs using two baits, and 16 645 SNPs using three baits, and 228 Y-chromosome targets representing sequence-tagged sites (STS), *AMLEY* and *SRY* genes. All 59528 Arbor myBaits were 80 nt long RNA probes.

For CNERs generation, we targeted the same randomly selected 22 619 autosomal SNPs, each with one 80-bp long CNERs centered at the SNP site, plus the same 228 Y chromosome targets. To test the effect of CNERs length on coverage, we selected two additional sets of 576 SNPs and designed 50 bp and 100 bp CNERs with SNPs at the center. In total, the horse SNP panel targets 23 771 SNPs using a total of 23 999 probes.

### Horse SNP panel CNERs generation

We generated CNERs for the horse SNP panel as schematically described in Figure 1. We appended six deoxy-T (dT) bases at the 5′ end, and AscI restriction site and (dT)_6_ at the 3′ end to all horse target regions to make CNERs templates. We synthesized the templates as an DNA oligo pool using silicon chip based phosphoramidite chemistry (Twist Biosciences). We circularized 100 or 300 femtomoles of the oligo pool in a 20 μl splint ligation reaction containing 2000 U T4 DNA ligase (NEB), 10 U T4 PNK (NEB) and 1000 fmol (dA)_12_ splint oligo in 1× T4 DNA ligase buffer at 37°C for 1 h followed by 25°C for 3 h and denatured at 95°C for 3 min. We amplified the circularized oligo pool in a 50 μl RCA reaction containing 30U of Phi29 polymerase (NEB), 25 pmol each of forward (5′-AAAAAAAAAGGCGCGCC-3′) and reverse (5′-GGCGCGCCTTTTTTTTT-3′) RCA primers, 2 nmol each of biotin-11-dATP (Perkin Elmer) and biotin-11-dUTP (Thermo Fisher), 25 nmol each dNTPs in 1X Phi29 buffer with BSA. After 40–48 h of RCA reaction at 30°C, we purified RCA products using SPRI beads (1.2× ratio) and digested with 100 U AscI (NEB) for 5 h at 37°C to produce monomeric CNERs. We estimated size and concentration of RCA products before and after AscI digestion using capillary electrophoresis in a Fragment Analyzer (Agilent) with the genomic DNA kit. We purified post-digestion products using SPRI beads (2× ratio) and quantified the DNA using a Qubit (Thermo Fisher).

### CNERs hybridization capture optimization

We optimized CNERs capture for adapter blocker concentration, CNER amount per reaction, and hybridization buffer compositions. To optimize adapter blocker concentration, we titrated oligonucleotide blockers at 5×–200× molar excess to 100–300 ng (1.0–2.3 pmol) of the modern horse libraries, 25 ng horse SNP panel CNERs, 2.5 μg of Human c0t DNA, and 25 μg of salmon sperm DNA in 25 μl reaction, and then denatured at 95°C for 10 min. We added this DNA mixture to 25 μl prewarmed Hyb buffer (final concentrations: 6× SSPE, 6× Denhardt's solution, 10 mM EDTA, pH 8.0, 0.2% SDS) and hybridized the mixture overnight in 50 μl total reaction volume at 65°C. To optimize CNERs amount titrations, we hybridized 300 ng of libraries with 30–90 ng of horse SNP panel CNERs and 200x molar excess oligo blockers in the Hyb buffer at 65°C overnight. We tested four hybridization buffers (HB1: 100 mM MES pH 6.5 and 1 M NaCl; HB2: 6× SSC, pH 7.0; HB3: 6× SSPE, pH 7.4; and HB4: 100 mM Tris pH 8.0 and 1 M NaCl) to capture 250 ng of libraries using 50 ng CNERs overnight at 65°C. All four buffers also contained 0.1% SDS, 10 mM EDTA and 10% DMSO at final concentration. We captured CNER hybridized libraries onto 30 μl MyOne C1 streptavidin beads (Thermo Fisher) at 65°C for 30 min. We washed beads three times in high stringency wash buffer (0.2× SSC, 0.1% SDS and 10% DMSO) for 5 min each at 65°C and then three times in low stringency buffer (2× SSC and 0.1% SDS) at room temperature. We washed beads in 10 mM Tris pH 8.0 before resuspending in the PCR reaction. We amplified post-captured libraries using 2× KAPA HiFi master mix (Roche) and Illumina universal amplification primers at 98°C for 3 min, followed by 15 cycles of 98°C for 30 s, 60°C for 30 s, 72°C for 30 s, with a final extension at 72°C for 5 min then hold at 12°C. We purified post-capture libraries with 0.9× SPRI beads, quantified using a Qubit (Thermo Fisher), pooled, and sequenced on an Illumina NextSeq using PE 2 × 150 kit.

### Ancient horse DNA capture and sequencing

For the ancient horse samples, we captured 5 μl (constant library volume with varying library mass; see Supplementary Table S2 for details) of individual ancient horse libraries using Arbor myBaits and CNERs. For both Abor myBaits and CNERs captures, we performed two experiments. In experiments A1 (CNERs) and A2 (Arbor myBaits), we followed the Arbor myBaits protocol and used 50% of capture beads for post-capture amplification and purified libraries with 1.7× SPRI as per the protocol. In experiments B1 (CNERs) and B2 (Arbor myBaits), we followed the optimized CNERs protocol, and used 100% of capture beads for PCR and 0.9× SPRI for cleanup. Finally, we performed a separate CNERs Experiment C, in which we captured libraries in 3-plex pools. In experiment C, we also used 100% of captured beads for PCR amplification and purified the post-capture libraries with 0.9× SPRI.

For all experiments using CNERs, we used 2 μl (∼40 ng) of the horse SNP panel CNERs. For a single sample, UAM:ES:27502, for which little material remained at the start of the experiment, we used only 2 μl of library CNERs in both experiment A and B. For all other samples, we used 5 μl libraries for captures. We added 200× adapter blocking oligos, 2.5 μg of Human c0t DNA and 25 μg of salmon sperm DNA to these library-CNERs to a total of 30 μl volume, and then denatured at 95°C for 10 min. We preincubated 30 μl of HB4 at 62°C for 5min, mixed with denatured library/CNERs/blockers mixture and hybridized at 62°C for 19.5 h. We enriched post-hybridization libraries onto streptavidin beads as in the optimization experiments except both low and high stringency wash steps were done at 65°C.

For CNERs experiment C (pooled capture), we hybridized 67–100 ng of libraries for each of three samples with similar endogenous content with 40–60 ng CNERs (Supplementary Table S2). We repeated the individual capture for UAM:ES:26433, rather than including it in a pool, as it had the lowest pre-capture endogenous content. We did not perform pooled captures for Arbor myBaits as it was not recommended by the manufacturer.

For all captures using Arbor myBaits, we used 5 μl of the same ancient horse libraries that we used in CNERs captures. We used unopened vial of the Arbor myBaits Horse SNP panel. Although the baits had been stored at -80°C continuously since production, they were 15 months older than the labeled use-by date. We followed Arbor Biosciences capture protocol v3 with recommended modifications of hybridization at 55°C for 41 h for ancient DNA.

We used different approaches to post-capture library amplification in experiments A compared to experiments B. In A, we resuspended capture beads in 30 μl 10 mM Tris pH 8.0 buffer. We then used 15 μl of the resuspended beads in 20 cycles of PCR amplification with 2× KAPA HiFi. We then purified the product with 1.7× SPRI, as recommended by Arbor. For B, we resuspended capture beads in 20 μl 10 mM Tris pH 8.0 buffer and used all of it in a 50 μl PCR reaction and performed 20 cycles of amplification, followed by purification with 0.9× SPRI.

All post-capture libraries were Qubit (Thermo Fisher) quantified, pooled, and sequenced on an Illumina NextSeq with a PE 2 × 75 kit.

### Bioinformatic processing

We trimmed adapter sequences from the reads and merged overlapping paired end reads using SEQPREP2 (https://github.com/jeizenga/SeqPrep2). We mapped merged and unmerged reads to the EquCab2 reference (23) genome using BWA*aln* (version 0.7.17-r1188, 28). We marked and removed duplicated reads using Picard *MarkDuplicates* - v2.21.7 and calculated capture metrics using Picard *CollectHsMetrics* (version 2.21.7, http://broadinstitute.github.io/picard). We determined read coverage at target SNPs using bedtools *multicov* (version 2.29.1). We plotted SNP coverage against CNERs length, GC content, and percent targets using custom python scripts (https://github.com/bsun210/CNERs_ancient_horses). We used bedtools *intersect* (version 2.29.1) to find sequence reads mapping to the target SNPs to calculate the position of SNPs relative to the sequence read insert size. We determined genotype likelihoods for the ancient horses using ANGSD (version 0.935–52-g39eada3) with *-GL 2 -minMapQ 20 -nThreads 24 -doGlf 2 -doMajorMinor 1 -SNP_pval 1e-6 -doMaf 1* options (29). We analyzed population clustering and ancestry using PCANGSD (version 1.10) with default settings (30). We used *prcomp* and *factoextra* R packages (Kassambara. A and Mundt. F. (2020) Factoextra: Extract and Visualize the Results of Multivariate Data Analyses. https://cran.r-project.org/web/packages/factoextra/index.html) for principal component analysis (PCA). We calculated endogenous content (proportion of unique reads aligned to the horse genome), library complexity (proportion of uniquely-mapped non-duplicated molecules) and insert size distribution using the pipeline described in (15).

We assessed whether the SNP coverage for CNERs with different lengths, changes in endogenous content, library complexity, and insert size between pre and post-capture libraries are normally distributed using the Shapiro-Wilk test. All these groups are not normally distributed; hence we performed a nonparametric Mann-Whitney Wilcoxon (MWW) rank test for comparison between groups. For comparison of normalized coverage distribution across GC bins for various experimental groups, we used two sample Kolmogorov-Smirnov (KS) tests for goodness of fit.

## RESULTS

The CNER method is designed to generate large amounts of biotinylated baits for hybridization capture (Figure 1). CNER templates are synthesized as oligonucleotides with oligo-dT linkers at both 5′ and 3′ ends to facilitate circularization using a complementary, oligo-dA splint. Because the linkers are oligo-dT, this design limits the impact of incomplete oligonucleotide chemical synthesis errors at the template ends. In the 3′ end upstream of the oligo-dT, a rare-cutter restriction enzyme recognition site (RES) is also incorporated (Figure 1). Oligo-dT and rare cutter RES are appended to all target sequences such that all CNER templates have uniform ends to facilitate bulk circularization by splint ligation using an oligo-dA splint adapter (Figure 1).

After circularization, CNER templates are bulk amplified by rolling circle amplification (RCA) using high processivity phi29 DNA polymerase. The RCA reaction includes biotin-dATP and biotin-dUTP (an inexpensive and widely available alternative for biotinylated dTTP) in the reaction to generate biotinylated products. An oligo-dA forward primer and oligo-dT reverse primer initiate forward and reverse RCA reactions. Thus, the RCA products for each CNER template is double-stranded, regardless of which strand the original CNER template was designed against (Figure 1). This conveniently generates probes against both strands of each CNER targeted region. Further, inclusion of both forward and reverse primers facilitate branched amplification during RCA to increase yield. The RCA makes many of copies of the CNERs as concatemers, a single restriction enzyme digestion of which produces monomeric, biotinylated capture probes (Figure 1). The monomeric CNERs can therefore be used as baits to capture and enrich target molecules on streptavidin-coated beads for sequencing.

We designed a horse SNP panel with 23 771 randomly selected SNPs from a list of high confidence variant sites ascertained in four ancient horse genomes (15). Chemical synthesis of oligo templates for this panel yielded a 215 ng (6.3 pmol) pool. RCA amplification of 100 femtomoles (∼3.3 ng) bulk circularized template pool generated 611 ng of double-stranded high-molecular weight DNA (∼77 kB average size, Supplementary Figure S1A), restriction digestion of which generated 499 ng of monomeric CNERs with 114 bp average size (Supplementary Figure S1B). The presence of double-stranded DNA indicates that the CNERs method generates probes against both strands of the target region. In a separate experiment, we increased the input template to 300 femtomoles. The protocol yielded 1.57 μg CNERs in that experiment. Thus, we estimate 100 fmol (∼3.3 ng) of circularized CNER templates produces ∼500 ng of CNERs using the protocol as described.

### CNER hybridization optimization

**Figure 2.**
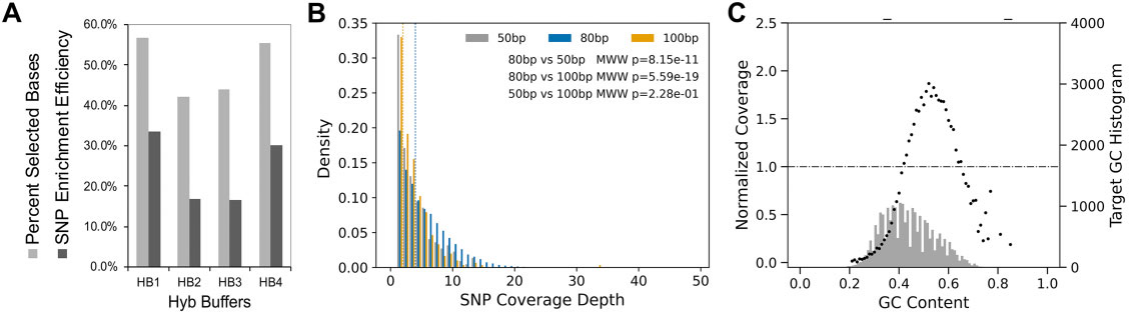
Optimization of CNERs hybridization capture of SNPs in four modern horse samples. (**A**) Enrichment efficiency for four hybridization buffers with pH varying from 6.5 to 8.0 (HB1 - 4). Light grey bars show the Percent Selected Bases determined using Picard tools and dark grey bars show the SNP enrichment efficiency. Values presented are the average of three experiments for HB1 and HB4 buffers and exact values for a single experiment for HB2 and HB3. (**B**) Histogram density plots of SNP coverage depth for three CNER lengths. SNPs captured with 80bp CNERs (blue bars) result in significantly higher coverage compared to SNPs captured with 50 bp (grey bars) or 100 bp (orange bars) CNERs; p-value is from a Mann–Whitney Wilcoxon test. Dotted lines indicate the mean coverage for each CNERs length. (**C**) Mean of normalized coverage (primary Y-axis) plotted across GC content of CNER target regions show that regions with 43–65% GC have sample-normalized coverage of 1 or higher. A histogram of GC bins across the target regions is shown in the secondary Y-axis.

We optimized in-solution hybridization conditions for the horse SNP panel CNERs using the modern horse DNA libraries (see Supplementary Data). We tested hybridization capture reactions with increasing amounts of adapter blocking oligos to prevent cross-hybridization of library molecules (31) with a constant amount of CNERs. In a separate set of experiments, we tested increasing amount of CNERs with a constant amount of blocking oligos. Both increasing amount of blocking oligos and CNERs modestly improved the enrichment efficiency (Supplementary Table S3, Supplementary Figure S2A and B). We note that conventional hybridization buffer like those used by Arbor myBaits for RNA baits (32) might be suboptimal for DNA baits. Therefore, we tested four hybridization buffers (HB) to improve the enrichment efficiency for CNERs. Captures in HB4 produced >50% (by Picard metrics) bases on or near targets for the modern horse libraries (Figure 2A). Additives used in conventional hybridization buffers like Denhardt's solution and trimethyl ammonium chloride did not improve and or lowered the percentage of on or near target bases (Supplementary Figure S2C). Hybridization at 62°C and 65°C also resulted in similar enrichment efficiency (Supplementary Figure S2D).

Existing capture bait synthesis methods use different probe lengths and tiling to optimize for the GC content of target regions (33, 34). We designed CNERs with three different lengths to test the effect of CNER length on SNP coverage. The 80 bp CNERs produce higher SNP coverage than either 50 bp or 100 bp CNERs (Figure 2B) consistently across various hybridization conditions (Supplementary Figure S3). Further, target regions within 43–65% GC bins, which are 47% of the total target SNP regions (average GC = 43.8%), consistently resulted in ≥1 normalized coverage (Figure 2C, Supplementary Figure S4).

### CNERs efficiently capture ancient DNA target SNPs

**Figure 3.**
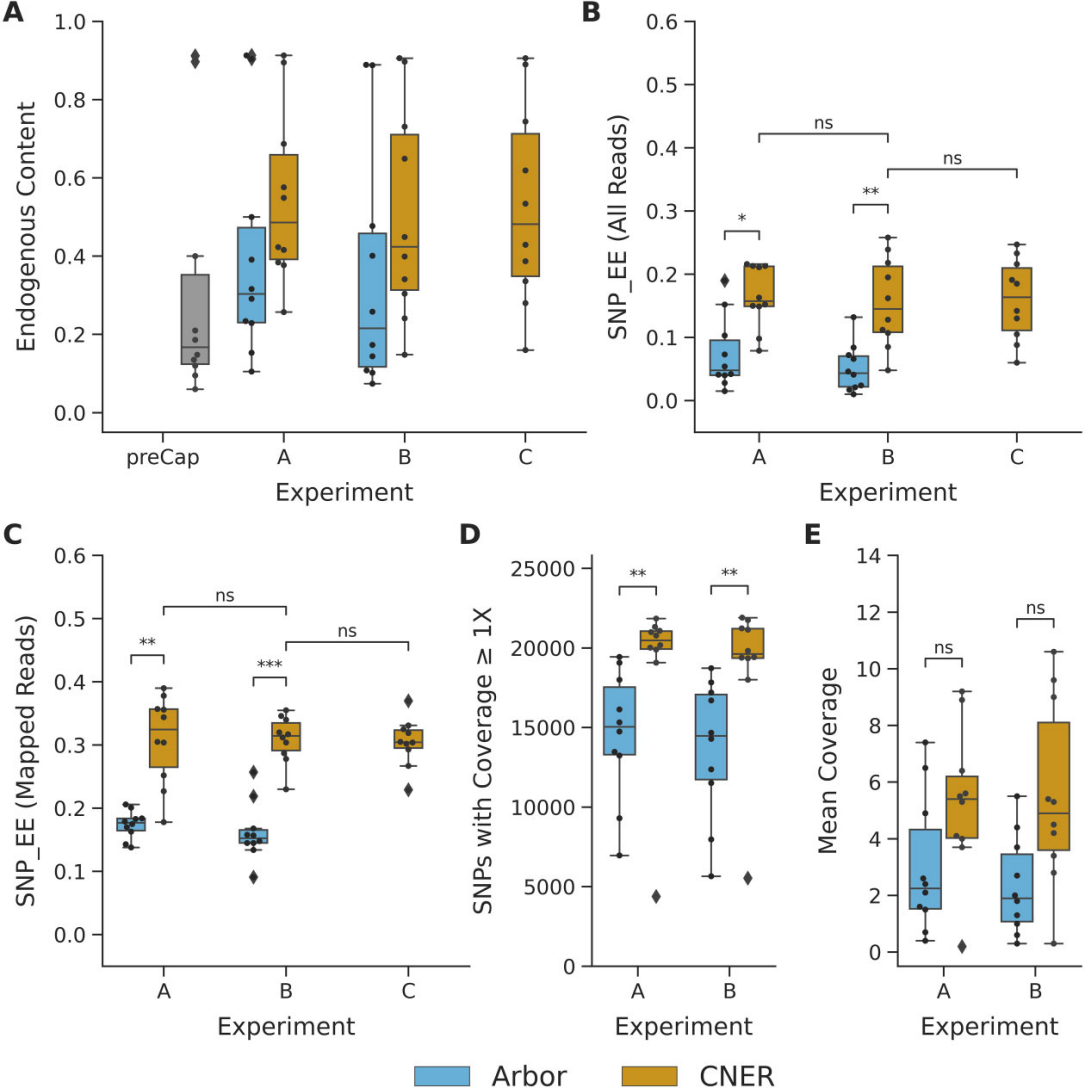
SNP capture with CNERs and Arbor myBaits for ancient horse samples. (**A**) Endogenous content measured as proportion of reads mapping to horse reference genome for ten ancient horse samples before capture enrichment (grey bars), proportion of mapped reads after capture with Arbor myBaits (cyan), and proportion of mapped reads after capture with CNERs (yellow). SNP enrichment efficiency measured as proportion of total reads (**B**) and mapped reads (**C**) covering the target SNPs for CNERs and Arbor myBaits. (**D**) Number of target SNPs covered by at least one read. (**E**) Mean coverage of target SNPs at one million raw read pairs. Mann–Whitney Wilcoxon test *P* values are indicated as ns (5.00e-02 < *P* ≤ 1.00e + 00), * (1.00e-02 < *P* ≤ 5.00e-02), ** (1.00e-03 < *P* ≤ 1.00e-02) and *** (1.00e-04 < *P* ≤ 1.00e-03).

We extracted DNA from ten horse bones collected from Late Pleistocene age permafrost deposits in Alaska, USA and Chukotka, Russia (Supplementary Table S1 and (15)). Sequence reads generated from each of these samples, mapped to the EquCab2 reference genome, provided estimates of endogenous DNA content. Before SNP enrichment, the ancient horse DNA libraries had 18.4% median reads mapped to the horse genome, across a wide range (6.0–91.2%, ‘preCap’ in Figure 3A, Supplementary Table S2).

SNP enrichments using both DNA based CNERs and RNA based Arbor myBaits increased the proportion of reads in the sequencing library that mapped to the reference genome, indicating successful target enrichment. Enrichment using CNERs improved median precent of mapped reads to 37.9% in experiment A (individual captures following the Arbor myBaits protocol), and 30.5% in experiment B (individual captures following the CNERs protocol), and 40.1% in experiment C (pooled-captures with CNERs protocol). Arbor myBaits resulted in 28.8% in experiment A (individual capture following the Arbor myBaits protocol), and 21.1% in experiment B (individual capture following the CNERs protocol) (Figure 3A, Supplementary Table S4. Comparison of CNERs experiments B versus C show a consistent proportion of mapped reads when a sample was captured individually versus as part of a pool (Figure 3A). The differences between capture probes and protocols are not significant by Mann–Whitney Wilcoxon test.

Different SPRI bead ratio used in the post-capture purification steps did not affect the proportion of mapped reads (Figure 3A). However, the different SPRI ratio resulted in different proportions of merged and unmerged reads identified during data analyses. Short insert size of aDNA molecules result in overlapping read pairs which are merged during data processing, hence called as merged reads. Read pairs that did not overlap are processed as unmerged read pairs. Following the Arbor myBaits protocol which uses 1.2x SPRI beads ratio (experiments A) resulted in a higher proportion of merged compared to unmerged reads for both Arbor myBaits and CNERs (Supplementary Figure S5A, Supplementary Table S4). All experiments that followed the CNERs cleanup protocol resulted in equal proportions of merged and unmerged reads regardless of probes, due to the lower SPRI beads ratio (0.9×) used during the post-amplification cleanup. Across all experiments, a greater proportion of merged reads mapped to the reference genome compared to unmerged reads, as expected for aDNA (Supplementary Figure S5B).

Previous studies used Picard's program *CollectHsMetric* to measure the success of target enrichment (35). This tool reports coverage of the targeted base and 100bp flanking regions when determining ‘Percent Selected Bases’. We used this metric during the optimization experiments to compare the performance of CNERs to current standards. However, this metric overestimates the SNP enrichment success by including the regions around the target SNP site. Therefore, we elected to measure the success of SNP enrichment in ancient horses by defining ‘SNP enrichment efficiency’ as the percentage of all or mapped reads that overlap the target SNPs. This is a straightforward and more practically important measure of SNP enrichment success. For the modern horse captures with CNERs, hybridization in HB4 at 65°C for 18–20 h produced ∼30% SNP enrichment efficiency for mapped reads (Figure 2A). We followed these hybridization conditions to capture ancient horse samples.

SNP enrichment efficiency, or the proportion of reads mapping to the target SNPs, was significantly higher when using CNERs compared to when using Arbor myBaits. In experiments A (Arbor myBaits protocol), the median SNP enrichment efficiency was 15.7% for CNERs versus 4.8% for Arbor (MWW *P* < 0.05). In experiments B (CNERs protocol), the median SNP enrichment efficiency was 14.5% for CNERs versus 4.3% for Arbor myBaits (MWW *P* < 1e-2; Figure 3B, Supplementary Table S4). This pattern holds when considering only reads that map to the reference genome. Experiments A (Arbor myBaits protocol) resulted in median enrichment efficiencies of mapped reads of 32.4% for CNERs versus 17.7% for Arbor myBaits (MWW *P* < 1e-2), and experiments B (CNERs protocol) resulted in median efficiencies of mapped reads of 31.5% for CNERs versus 15.2% for Arbor myBaits (MWW *P* < 1e-3; Figure 3C). The pattern is also consistent when considering merged and unmerged reads separately, both for all reads and mapped reads (Supplementary Figure S5C and D), although unmerged reads always had significantly lower enrichment efficiency compared to merged reads (Supplementary Figure S5D, Supplementary Table S4). Finally, the enrichment efficiency when using CNERs was consistent between individually captured libraries and captures performed in pools (Figures 3B and C).

To test the potential impact of differences in sequencing depth, we subsampled data to one million read pairs per sample in experiments A and B. For this analysis, we considered only the 22 619 target SNPs that were common between CNERs and Arbor myBaits. For experiments A (Arbor myBaits protocol), this read depth resulted in a median of 90.5% (20 479) of target SNPs covered by at least one unique read using CNERs versus 66.5% (15 038) for Arbor myBaits (MWW *P* < 1e-2; Figure 3D, Supplementary Table S4). We observed a similar trend when following the CNERs protocol (experiments B; Figure 3D). At this coverage, CNERs captures have fewer SNP dropouts compared to Arbor myBaits captures, as estimated using cumulative distribution plots of SNP coverage as percentage of SNPs less than the x-fold mean coverage (Supplementary Figure S6). When averaged across the 10 horse data sets at this standard coverage, CNERs captures resulted in 2.5-fold higher average SNP coverage than Arbor myBaits (an average of 5.4 reads per SNP compared to an average of 2.2 reads per SNP when using the Arbor myBaits protocol (experiments A; Figure 3E), and an average of 4.9 reads per SNP compared to an average of 1.9 reads per SNP when following the CNERs protocol (experiments B; Figure 3E). The average coverage was not significantly different by MWW test due to one outlier sample (UAM:ES:27502), which was the sample for which we had to reduce library volume going into CNERs captures and has low SNP coverage.

We evaluated target coverage uniformity using fold-80 base penalty, which estimates additional sequencing required to bring 80% of the zero-coverage targets to mean coverage depth. The smaller the fold-80 base penalty, the more uniform the coverage is across all target regions (36). The average fold-80 base penalty is 3.7 for CNERs and 5.3 for Arbor myBaits, suggesting that CNERs produces more uniform coverage across all target SNPs.

We explored whether probe length or GC content explained coverage unevenness among the ancient horses. As observed in the modern horse enrichments, enrichment of ancient horses resulted significantly higher SNP coverage for CNERs targeting 80bp regions compared to 50bp or 100bp (Supplementary Figure S7). The statistical degree of significance of these comparisons as estimated from MWW test p-values (Supplementary Figure S7) differed among the ancient horses due to differences in percent mapped reads. Enrichments using CNERs resulted in higher normalized coverage for SNPs in target regions that had 42–66% (mode ∼55%) GC content compared to SNP targets in other GC contents and to Arbor myBaits capture data in this GC bin (Supplementary Figure S8). Arbor myBaits resulted in higher SNP normalized coverage for target regions with 30–45% GC content (mode ∼37% GC) compared to other GC contents and to CNERs capture data in this GC bin. While this indicates a shift towards lower GC preference for Arbor myBaits and higher GC preference for CNERs, the difference in coverage across GC bins is not statistically different by KS test (Supplementary Figure S8).

We next compared CNERs captures and Arbor myBaits captures in the mean normalized coverage at 100 bp upstream and downstream regions of target SNPs to assess whether coverage around the SNP target region influenced coverage unevenness. We designed only one CNER per target SNP, centered in the target region, resulting in maximum coverage depth for SNPs and reduced coverage for the surrounding region (Supplementary Figure S9). Arbor myBaits designed up to three baits per target SNP, tiled 20 bp from 5′ end, which resulted in an expected maximum coverage for ∼20 bp region to the right of the target SNP (Supplementary Figure S9). These differences in coverage profile between CNERs and Arbor myBaits are significant by KS test.

Post-capture purification steps did not affect the coverage around SNPs; both experiments A (Arbor myBaits protocol) and experiments B (CNERs protocol) resulted in similar coverage profiles when comparing enrichments using same probes (Supplementary Figure S9).

### CNERs and Arbor myBaits produce similar genotypes

**Figure 4.**
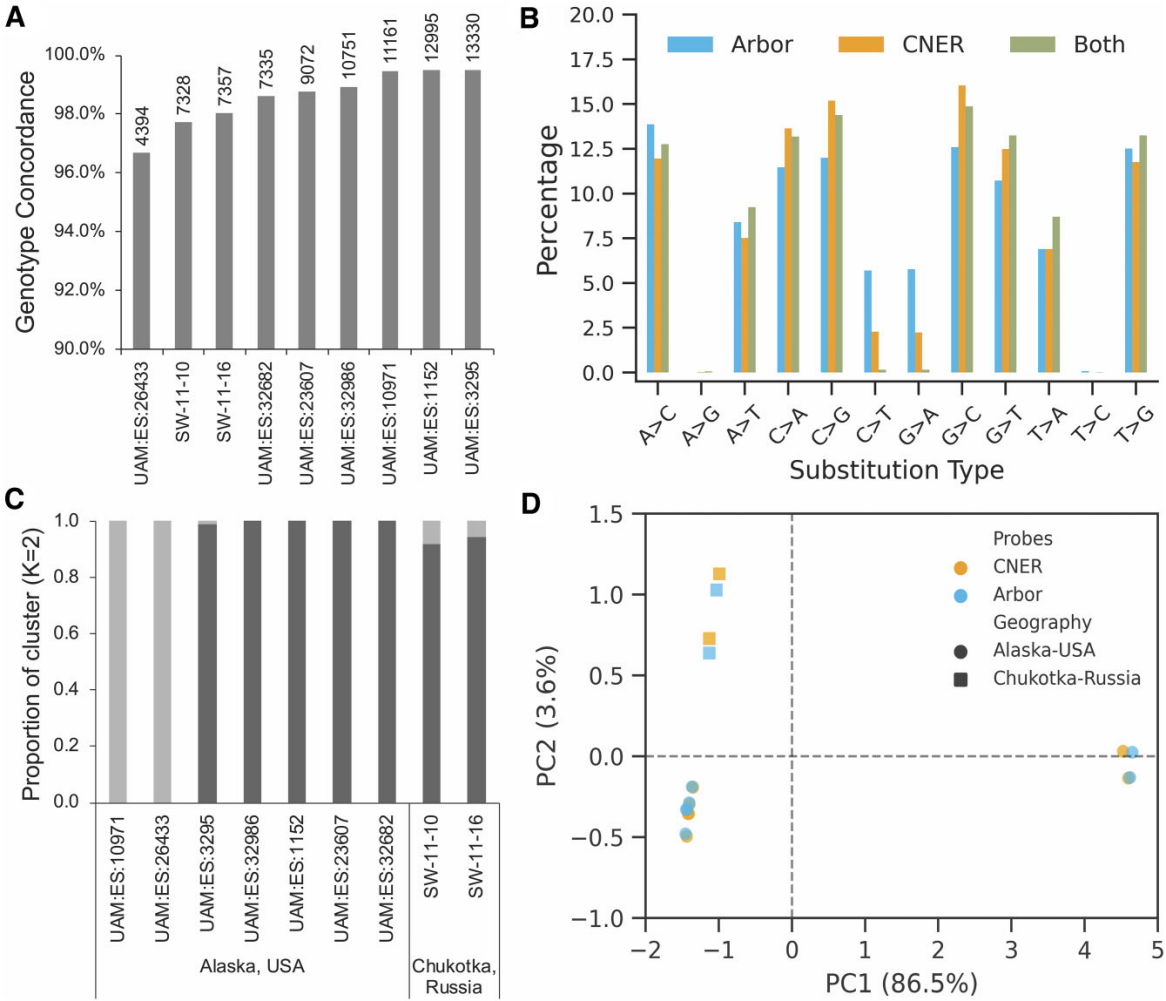
Genotyping and estimated evolutionary relationships between the ancient horse samples. (**A**) Genotype concordance between SNP capture data generated using CNERs and Arbor myBaits. Numbers above the bars indicate the sites genotyped by both methods in a given horse sample. (**B**) Percentage of substitution types shared between (green) and unique to CNERs (yellow) and Arbor myBaits (cyan). (**C**) Admixture analysis with *K* = 2 separated the ancient horses into two lineages regardless of their geographic location. (**D**) Principal component analysis of genotype likelihood covariance matrix of 23771 nuclear SNP sites in nine ancient horses. Transitions are filtered out for population analyses due to cytosine deamination in aDNA. PC1 segregated horses into two major clades and PC2 separated horses into the Western (Chukotka) and Eastern (Alaska) Beringian populations.

We calculated genotype likelihoods for target SNPs using the capture data. We did not include sample UAM:ES:27502 because it had few genotyped sites. Average concordance of genotypes of nine ancient horses between experiment A (Arbor myBaits protocol) and B (CNERs protocol) is 97.9% for Arbor myBaits data and 98.1% for CNERs data (Supplementary Figure S10, Supplementary Table S5). To increase the read depth for individual SNPs, we merged bam files from the two experiments and called genotypes on the merged data. With merged data, both CNERs and Arbor myBaits genotyped between 4394 and 13 330 sites with 96.7–99.5% concordance for individual horses (Figure 4A). On average, genotypes called on Arbor myBaits and CNERs data concur 98.6%.

CNERs and Arbor myBaits captured reads with different base substitution patterns in the target SNPs (Figure 4B). Of the total 18 994 genotyped sites among the nine ancient horses, 13 893 sites were captured using both probes, 1334 sites were only captured by Arbor myBaits and 3767 sites were only captured by CNERs data. CNERs capture more GC transversions compared to Arbor myBaits (Figure 4B) because they more efficiently capture higher GC regions (Supplementary Figure S8). While CNERs and Arbor myBaits capture reads with comparable patterns of cytosine deamination at the ends of reads (Supplementary Figure S11), Arbor myBaits captured more SNPs with transition substitutions (11.5% versus 4.5% for CNERs versus 0.4% shared in both probes, Figure 4B). This pattern may arise because the right shifted tiling design preferentially enriches for SNPs at the ends of aDNA molecules (Supplementary Figure S12) where transition substitutions occur due to cytosine deamination. Alternatively, CNERs enrich for aDNA fragments with SNPs at the center of the read (Supplementary Figure S12), which may lead to higher coverage at SNP sites compared to Arbor myBaits (Supplementary Figure S9).

We used the enriched genotypes to explore the evolutionary relationships between the nine ancient horses for which we generated data. Admixture analysis identified two main ancestry components, both for data generated using CNERs (Figure 4C) and Arbor myBaits captures (Supplementary Figure S13). Principal component (PC) analysis of genotype likelihood covariance also segregated ancient horses into two major clusters (Figure 4D), with similar patterns observed when using CNERs or Arbor myBaits data. The first principal component (PC1) roughly corresponds to ancestry as in Figure 4C, and PC2 reflects geographic origin either in Chukotka, Russia (Western Beringia) or Alaska, USA (Eastern Beringia). This pattern is consistent among probe types and with horse population structure previously inferred from whole-genome and mitochondrial data (15).

## DISCUSSION

Targeted sequencing can provide a cost-effective method for data generation for many comparative genomics applications, in particular when the samples of interest contain only trace amounts of degraded DNA. However, the high cost of producing hybridization baits hinders the widespread adoption of this approach. Our approach, which we call Circular Nucleic acid Enrichment Reagent method, reduces both the cost and time required for generation of microgram quantities of probes. Incorporation of poly-dT overhangs at both ends in the CNER template design overcomes end synthesis errors in long oligonucleotide baits. The length of the poly-dT limits the circularization of templates by splint ligation using the poly-dA oligo. Poly-dA mediated splint ligation ensures that only templates with a certain length of poly-dT are amplified by RCA, thus eliminating incompletely synthesized baits. These template design features and isothermal amplification using RCA overcome many of the artifacts induced by PCR amplification of template oligo pools like non-specific amplification and generation of heterogenous products (Twist Bioscience's technical note). Further, standard PCR amplification requires inclusion of specific primer binding sequences at the ends that increase oligo length (9) and may interfere with hybridization capture. Future comparison of the CNERs methods with other PCR-based oligonucleotide amplification methods would be useful to explore the role of amplification biases in hybridization efficiency.

We optimized the hybridization conditions for the CNERs which differed from conventional hybridization conditions used for RNA baits. Enrichments using CNERs reduces the hybridization time to overnight incubation (18 - 20 hours) instead of the 48–72 h required in conventional capture methods for degraded DNA (32,34). This increase in efficiency may be useful in clinical diagnostics. Further, conventional baits are designed with multifold tiling baits per target (33,34) to achieve uniform coverage across different GC regions, but still underperform for target regions with >50% GC content (33,35). We designed only one CNER tiling per SNP target region to save both CNERs production cost and sequencing cost. CNERs capture results in higher coverage for target regions with 45 - 75% GC content than regions with other GC contents, similar to other DNA baits (35), whereas Arbor myBaits produced higher coverage for regions with 30–45% GC, similar to other RNA baits (34,35). Difference in the AT/GC bonding strength might differently influence the melting temperature of DNA-RNA heteroduplex and double stranded DNA molecules, which could lead to the observed coverage differences between the DNA and RNA baits for target regions with different GC content. It would be interesting to test whether multi-tiling CNERs for target regions with lower GC content brings their coverage closer to the sample mean coverage. Multi-tiling and probe length also increase the coverage for regions around the targeted region (32,33). This might be desired for some applications like exome capture, but it will reduce the cost-effectiveness of genotyping-by-sequencing (GBS). CNERs achieve highest coverage at the target SNP sites compared to adjacent regions which is desired for GBS applications.

To demonstrate the utility of the CNERs approach for GBS, we genotyped ∼23k nuclear SNPs in ten ancient horses using both DNA based CNERs and a commercially available RNA baits from Arbor myBaits. We found that SNP enrichment efficiency using CNERs was consistent across most of our ancient samples, despite their variability in pre-enrichment precent mapped reads (endogenous content). Further, CNERs provided two-fold higher SNP enrichment efficiency compared to Arbor myBaits. CNERs required only one probe per target SNP and enriched a greater number of targeted sites with maximal read depth at the target SNP site. Two-fold higher enrichment efficiency could be due to enrichment of both strands of target regions by the CNERs probes compared to one targeted strand by RNA baits from Arbor. This could be tested using double stranded RNA baits (35). Both admixture and PC analysis of genotype likelihoods grouped the ancient horses into two major clusters (Figure 4), like the results based on whole genomes (15). Future work using the horse SNP panel with a more geographically and temporally extensive sampling of ancient horses will provide new insights into the history of movement and gene flow among Late Pleistocene horses.

Although we focused on generating data from individual horse bones, CNERs can also be used for targeted DNA capture and sequencing from other sample types that are difficult to genotype by conventional methods (37). Cell-free and circulating tumor DNA (cf/ctDNA) isolated from liquid biopsies, for example, can be used to identify mutation burden in cancer patients, disease carrier status, and for noninvasive prenatal testing (38). DNA isolated from environmental samples like water and air and from ancient sediments can be used to reconstruct present and past environments noninvasively (39). DNA isolated from single rootless hair can be used to solve forensic cases (40). All these sample types are preserved as highly fragmented DNA, however, and often in complex mixtures, where targeted capture using CNERs provides a straightforward approach to generating useful comparative data (41).

The CNER method can be extended to generate whole genome enrichment (WGE) probes. Genome fragments of a reference or related species can be circularized by bridge adapters to included restriction enzyme sites, amplified, and digested as in oligo templates to make WGE-CNERs. These would be a DNA alternative for the whole-genome in-solution capture (WISC) method's RNA baits (32). WGE is valuable when exploring an unknown organism or enriching a taxon in mixtures, as well as when analyzing aDNA samples with low endogenous content. WGE can also be used to generate low-coverage genomes of a few individuals for SNP ascertainment, from which a target SNP panel for population studies can be designed. We expect the CNER method may be adopted by future studies for various GBS and WGE applications.

## Supplementary Material

gkad460_Supplemental_Files

## Data Availability

All raw sequencing data generated for this project are submitted to the SRA database under BioProject accession number PRJNA785663.
