# Supplementary material for: A method to generate capture baits for targeted sequencing
Source: Nucleic Acids Res. 2023 Jun 1;51(13):e69. doi: 10.1093/nar/gkad460 (PMC10359599; doi:10.1093/nar/gkad460)
Supplement: gkad460_Supplemental_Files [file gkad460_supplemental_files.zip › NAR-00455-Met-K-2023_Revised_Supplementary_data.pdf]

A method to generate capture baits for targeted sequencing

Balaji Sundararaman[1,3*], Alisa O. Vershinina[2], Samantha Hershauer[1,3], Joshua D. Kapp[2] Shelby Dunn[2,3], Beth Shapiro[1,2,3,4] and Richard E. Green[1,4*]

Supplementary data

CNER hybridization optimization

We optimized in-solution hybridization capture conditions for the equine SNP panel CNERs using the feral horse DNA libraries. Picard tool *CollectHsMetrics*'s 'Percent Selected Bases' is a widely used measure of success of hybridization capture experiments for target enrichment of a few hundred base target regions in a genome. Percent Selected Bases is the percentage of total unique mapped bases uniquely mapped within and 250bp near the target regions. Counting the bases mapping on and near the ~80bp CNER target regions overestimate the success of enriching for SNPs targets. Therefore, we define 'SNP Enrichment Efficiency' as the percentage of all reads or mapped reads which are exactly mapped to the target SNP loci. This is a straightforward measure of success of SNP enrichment, but it is a conservative measure compared to the existing literature standards and must be noted when comparing our methods to others.

We tested the effect of adapter blocking oligos on capture efficiency as cross-hybridization to the adapter sequence present on all library molecules can reduce the percent selected bases (28). Blocking oligos titrated at 5x - 200x excess to the molar concentration of libraries in Hybridization (Hyb) buffer (6X SSPE, pH 7.4 with Denhardt's solution) linearly increased the percent selected bases (Supplementary Table S3, Supplementary Figure S2A) but did not improve beyond 34%. CNERs titrated at 30 - 90ng per capture reaction in Hyb buffer with 10% DMSO modestly improved the percent selected bases (Supplementary Figure S2B). Hyb buffer is conventionally used for in-solution hybridization captures using RNA baits (29) and might be suboptimal for DNA baits.

We tested four hybridization buffers with pH 6.5 - 8.0 to improve the capture efficiency using CNERs. Captures in HB4 (pH 8.0, full composition in Methods) produced over 50% percent selected bases for the feral horse DNA libraries captured using equine SNP panel CNERs (main Figure 2A). Additives used in traditional capture buffers like trimethyl ammonium chloride and Denhardt's solution lowered the capture efficiency for CNERs compared to buffers without additives (Supplementary Figure S2C). Hybridizing at 62°C and 65°C produced a similar efficiency when captured in HB4 with Denhardt's solution (Supplementary Figure S2D).

Supplementary Figures

Figure S1: CNERs generation for the Equine SNP panel.

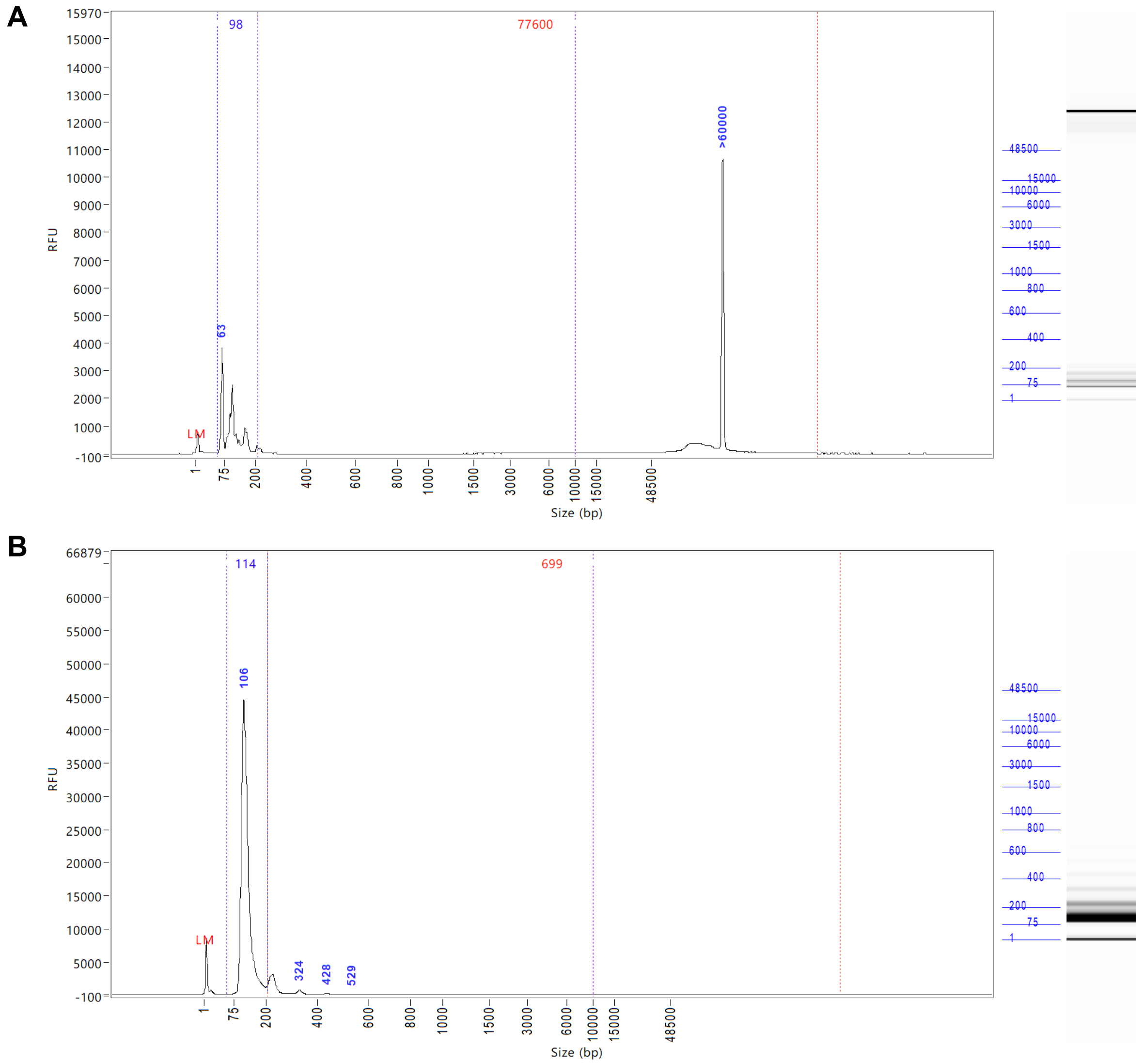

Figure S1: CNERs generation for the Equine SNP panel. (A) High molecular weight DNA with >60kb size made by RCA reaction are shown in the capillary electrophoretogram analyzed using Fragment Analyzer genomic DNA kit. (B) RCA products digested with AscI restriction enzyme generated >90% monomeric CNERs with 114bp mean size.

Figure S2: CNERs hybridization capture optimizations.

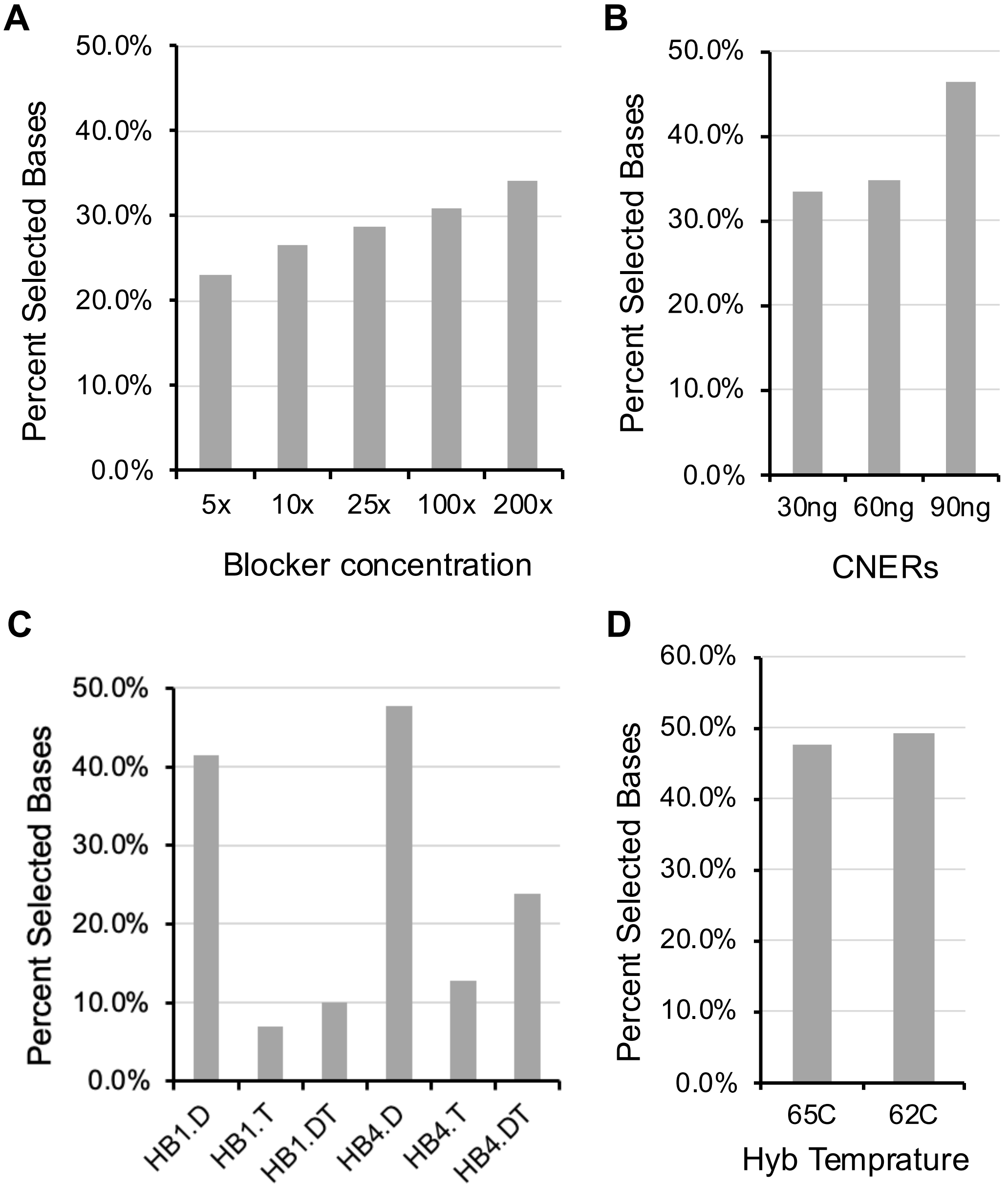

Figure S2: CNERs hybridization capture optimization. Increasing amounts of oligonucleotide blockers against Illumina adapters (**A**) and increasing amounts of CNERs per capture reaction (**B**) modestly improved the percent selected bases when performed in conventional hybridization buffer. **(C)** Trimethyl ammonium chloride ('T') and Denhardt's ('D') solution when added to HB1 and HB4 as additives, decreased the percent selected bases. **(D)** Hybridizations at 62C and 65C result in similar percent selected bases.

Figure S3: CNERs length affects SNP coverage.

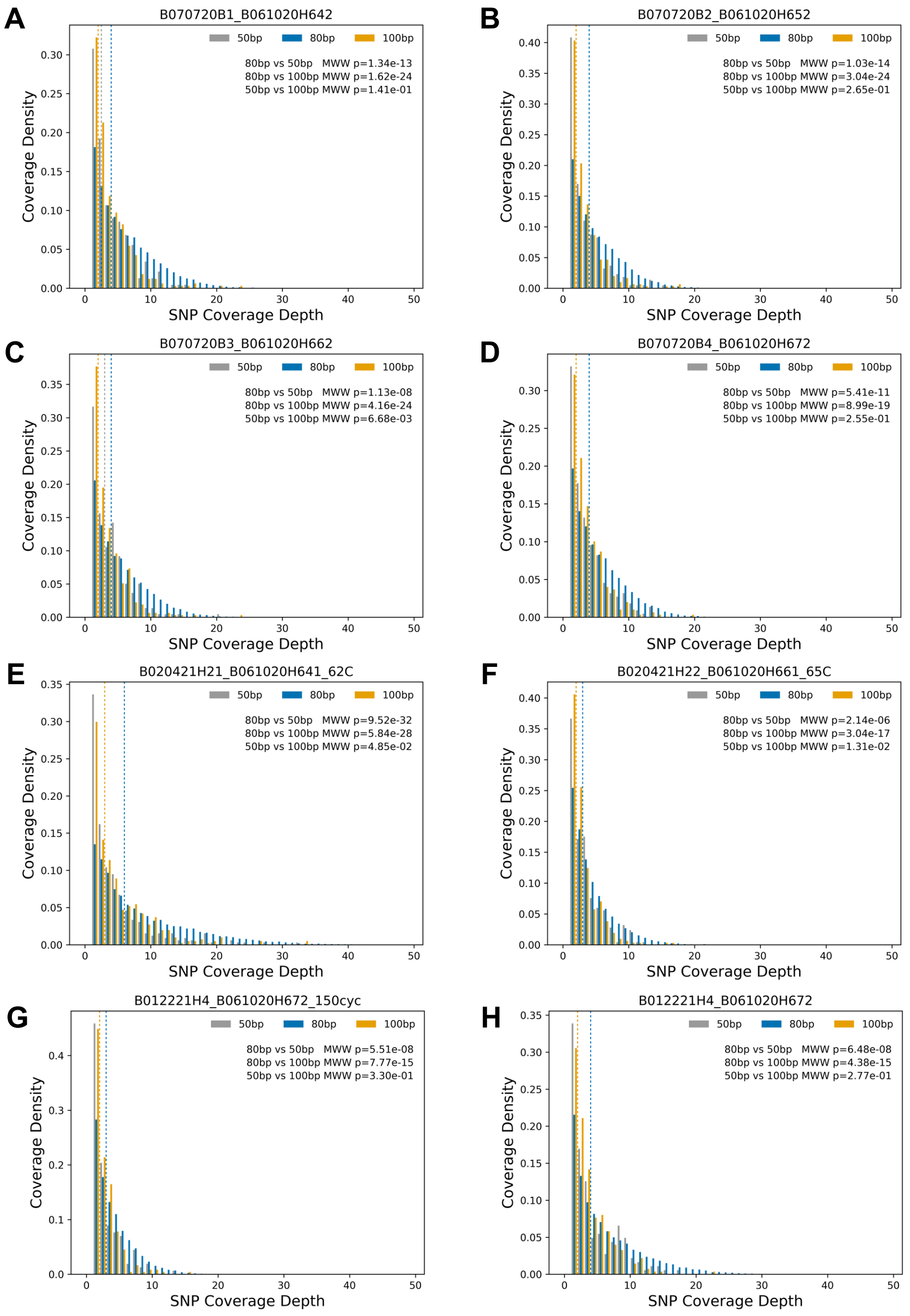

Figure S3: CNERs length affects SNP coverage. Overlapping histograms of the SNP coverage depth for 50bp (grey bars), 80bp (blue bars) and 100bp CNERs (orange bars). Median coverage for each group is shown in color-matched dotted vertical lines. The 80bp CNERs produce higher SNP coverage depth compared to both 50bp and 100bp CNERs, regardless of four hybridization buffers (**A-D**), temperature (**E and F**) and sequencing the same capture library for 150 cycles **(G)** or 300 cycles **(H)**. P values of the Mann-Whitney-Wilcoxon two-sided test with Bonferroni correction are significant for both 80bp vs 50bp and 80bp vs 100bp comparisons, but not always for 50bp vs 100bp CNERs.

Figure 4: SNP coverage across GC bins.

A

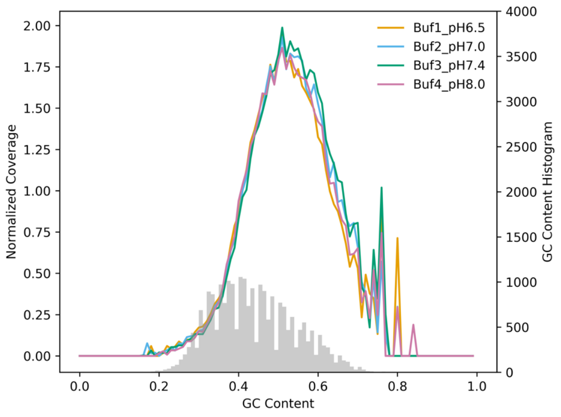

B

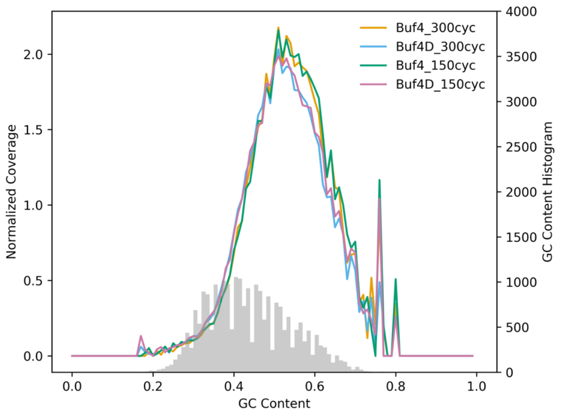

C

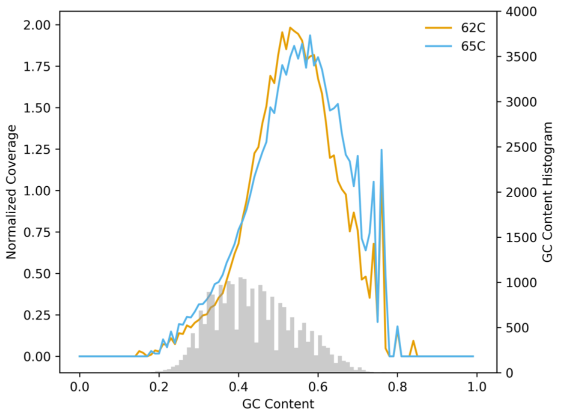

D

Kolmogorov–Smirnov test

Sample 1	Sample 2	KS stat alpha	KS stat p-value
Buf1_pH6.5	Buf2_pH7.0	0.03	1.0000
Buf1_pH6.5	Buf3_pH7.4	0.05	0.9997
Buf1_pH6.5	Buf4_pH8.0	0.05	0.9997
Buf2_pH7.0	Buf3_pH7.4	0.04	1.0000
Buf2_pH7.0	Buf4_pH8.0	0.04	1.0000
Buf3_pH7.4	Buf4_pH8.0	0.05	0.9997
Buf4_150cyc	Buf4D_150cyc	0.05	0.9997
Buf4_300cyc	Buf4D_300cyc	0.05	0.9997
Buf4_300cyc	Buf4_150cyc	0.04	1.0000
Buf4_300cyc	Buf4D_150cyc	0.05	0.9997
Buf4D_300cyc	Buf4_150cyc	0.06	0.9942
Buf4D_300cyc	Buf4D_150cyc	0.04	1.0000
62C	65C	0.06	0.9942

Figure S4: SNP coverage across GC bins. Mean of normalized coverage (primary Y-axis) plotted across GC content of CNER target regions show that regions with 43% - 65% produce sample-normalized coverage of 1 or higher. Histogram of GC bins across the target regions is shown in the secondary Y-axis. Coverage across GC bins is consistent for various hybridization buffers (**A**), capturing with or without Denhardt's solution and sequencing for 150 or 300 cycles **(B)** and hybridizing at 62C or 65C **(C)**. Pair-wise comparisons tested by the Kolmogorov-Smirnov two sample test show that the coverages are from the same distributions (**D**).

Figure S5: SNP enrichment efficiency differs between merged and unmerged reads.

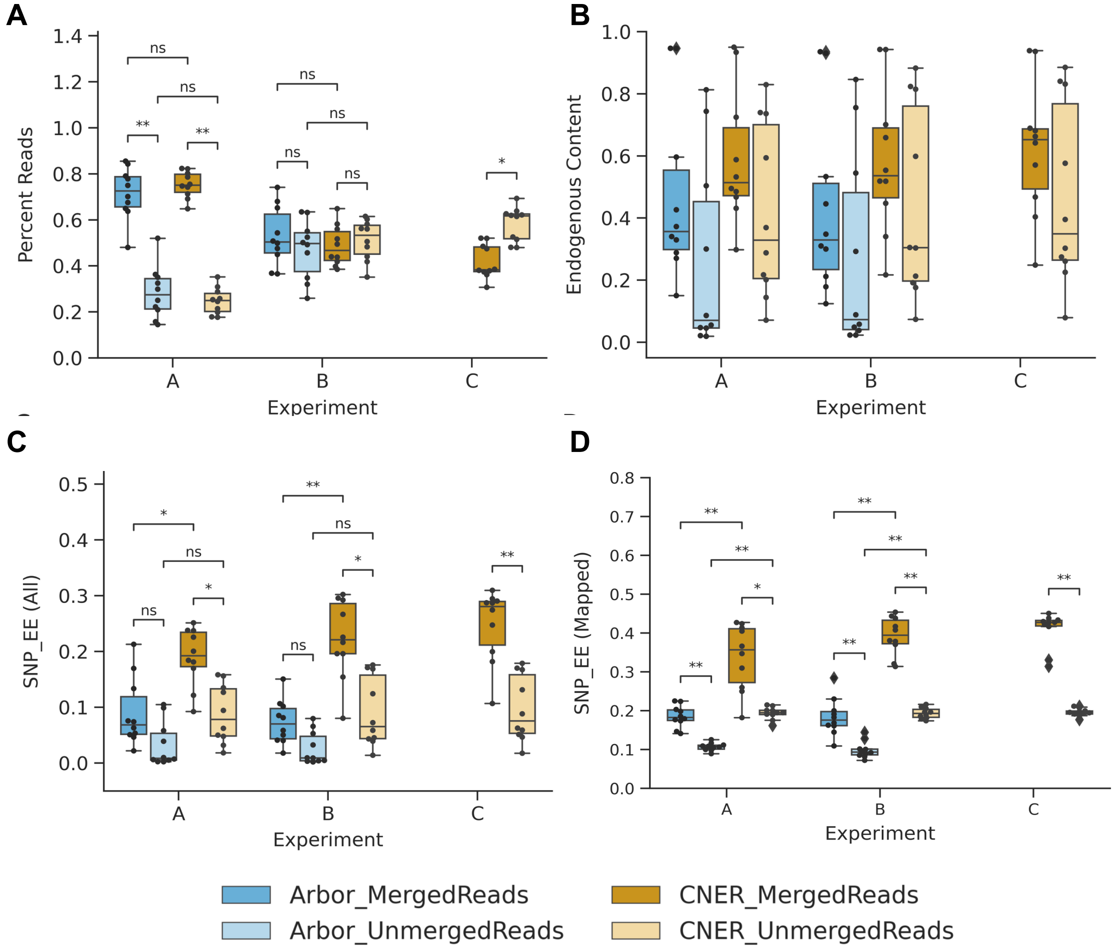

Figure S5: SNP enrichment efficiency differs between merged and unmerged reads. In experiment A, samples were individually captured by following the Arbor myBaits protocol. In experiment B, samples were individually captured by following the CNERs protocol. And in experiment C, samples were pooled before captures by following CNERs protocol. **(A).** Merged reads (dark shades of colors) is significantly higher than unmerged reads (light shades of colors) in experiment A which followed 0.9X SPRI bead purification for post-capture cleanup for both CNERs (yellow) and Arbor myBaits (cyan). Experiments B and C have about equal proportion of merged and unmerged reads for both probes. **(B)** Merged reads produced higher endogenous content than unmerged reads across all experiments and with both probes. (**C)** CNERs produced ~2-fold higher SNP enrichment efficiency in all reads group when merged and unmerged reads looked individually. There is no difference in the capture efficiency between two read groups for same probe. (**D)** In the mapped reads, unmerged reads produced significantly lower enrichment efficiency across all experiments and probes. CNERs consistently produced >2-fold higher efficiency than Arbor myBaits when merged and unmerged mapped reads looked individually. Mann-Whitney U test p values are indicated as ns (5.00e-02 < p <= 1.00e+00), * (1.00e-02 < p <= 5.00e-02), ** (1.00e-03 < p <= 1.00e-02) and *** (1.00e-04 < p <= 1.00e-03).

Figure S6: Cumulative SNP coverage distribution plots.

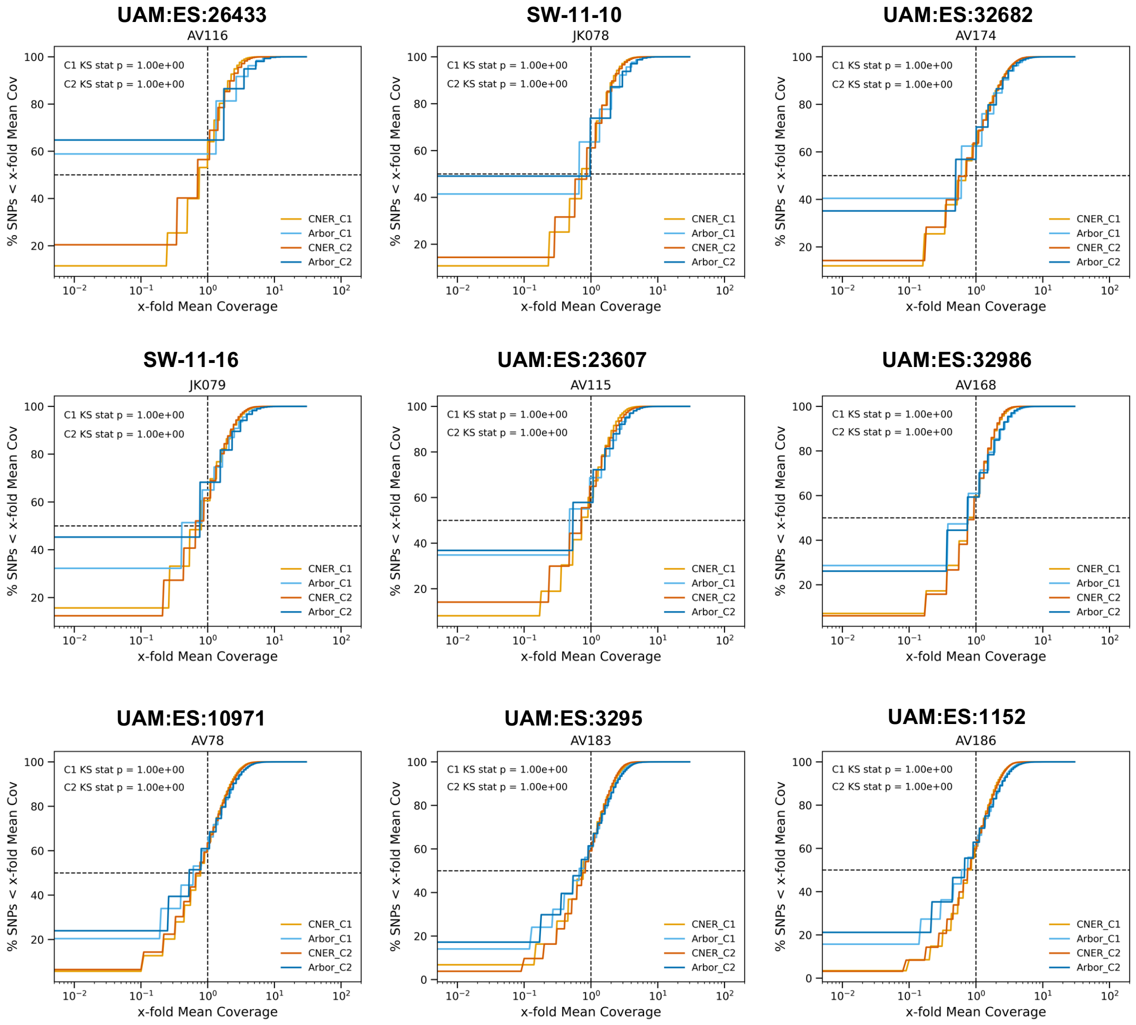

Figure S6: Cumulative SNP coverage distribution plots. Samples were individually captured by following the Arbor myBaits protocol in experiment A (labeled as ‘C1’) and by following CNERs protocol in experiment B (labeled as ‘C2’). Plots show cumulative percentage of targeted SNPs (y-axis) that have coverage below x-fold mean coverage (x-axis) for nine ancient horse captures with CNERs (yellow lines) and Arbor myBaits (cyan lines). Y-intercept shows the percentage SNP dropouts (SNPs with zero-coverage). CNERs have lower SNP dropout rate (y-intercept) than Arbor myBaits. Experiments A (labeled as ‘C1’) and B (labeled as ‘C2’) have identical coverage distribution when compared for the same probes.

Figure S7: 80bp long CNERs result in higher SNP coverage for ancient samples.

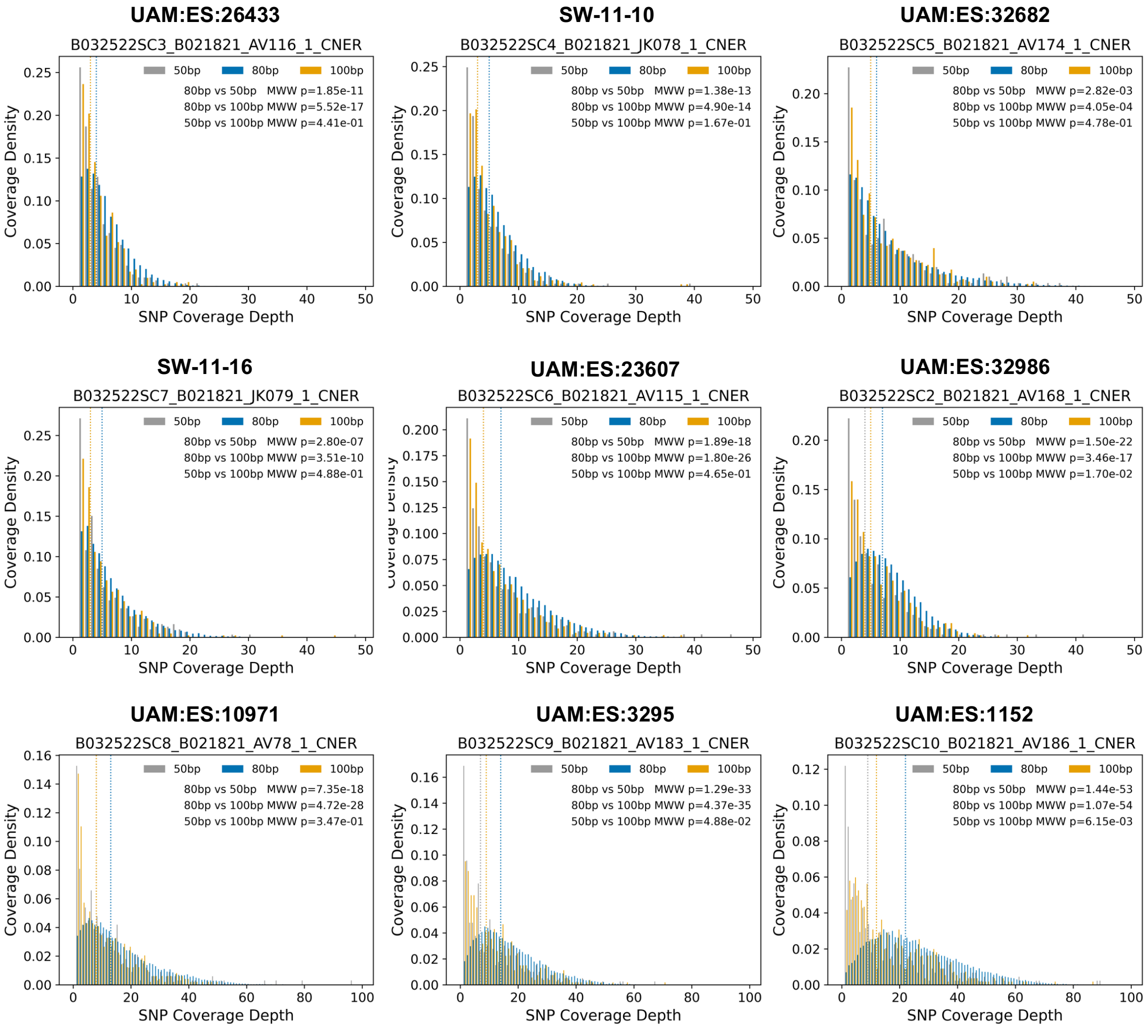

Figure S7. 80bp long CNERs result in higher SNP coverage for ancient DNA samples similar to modern feral horse DNA experiments. Median coverage noted by the color matched dotted vertical lines. Difference in overall sequencing depth due to differences in endogenous contents led to varying degrees of significance (Mann-Whitney-Wilcoxon P values).

Figure S8: SNP coverage across GC bins for ancient samples.

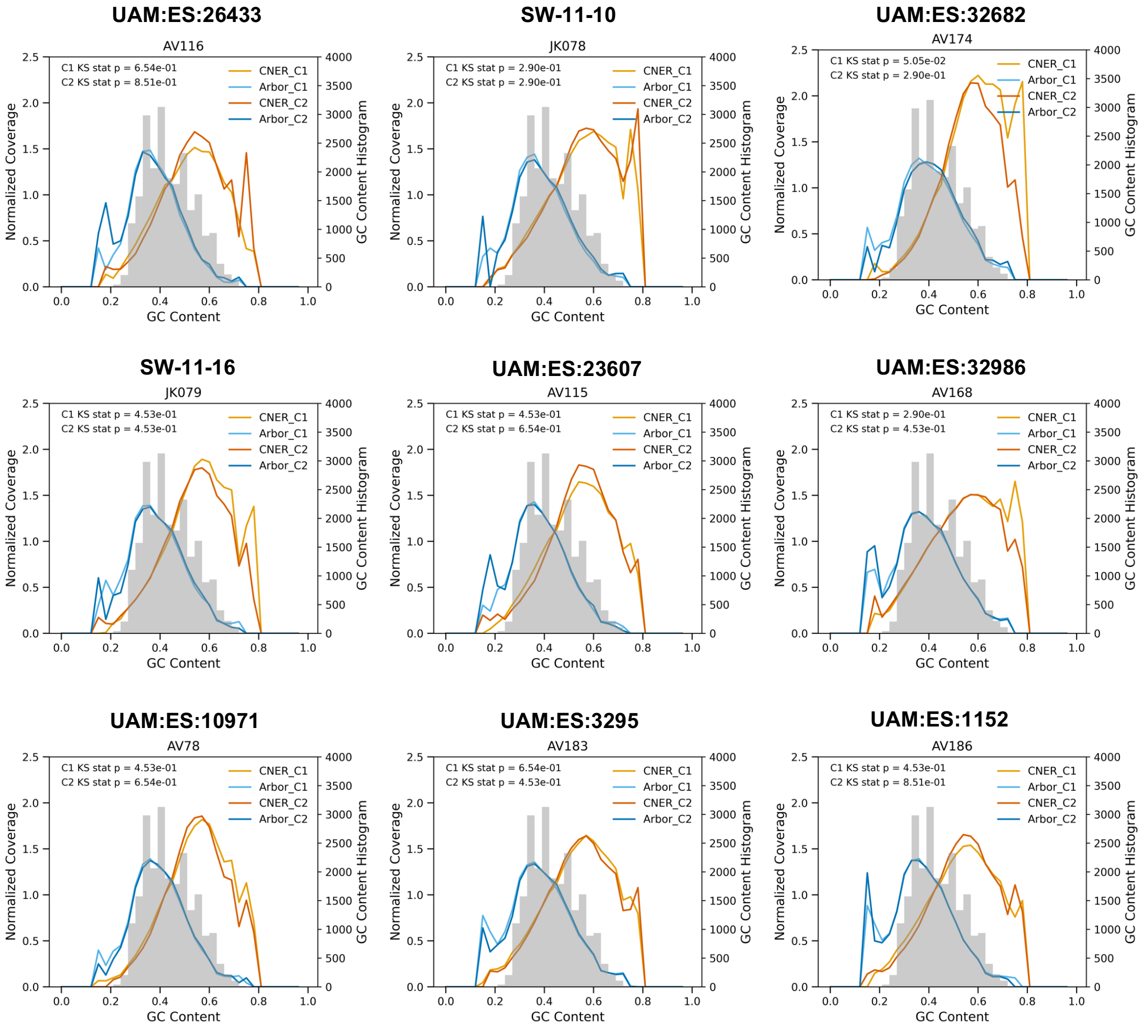

Figure S8: SNP coverage across GC bins for ancient samples. Samples were individually captured by following the Arbor myBaits protocol in experiment A (labeled as 'C1') and by following CNERs protocol in experiment B (labeled as 'C2'). Normalized coverage (y-axis) across GC bins (x-axis) shows that CNERs (yellow lines) for target regions with 43% - 65% GC produce sample-normalized coverage of 1 or above for ancient DNA samples similar to modern feral horse DNA experiments. Arbor myBaits produced >=1 normalized coverage for target regions with 30 – 45% GC. GC histogram of all target regions is shown in secondary y-axis (grey bars). Experiments A (labeled as 'C1') and B (labeled as 'C2') have identical coverage distribution when compared for the same probes. KS test between CNERs and Arbor myBaits show no significant difference in the GC coverage profile for both experiments (C1 and C2).

Figure S9: CNERs result in maximal coverage depth at SNP sites.

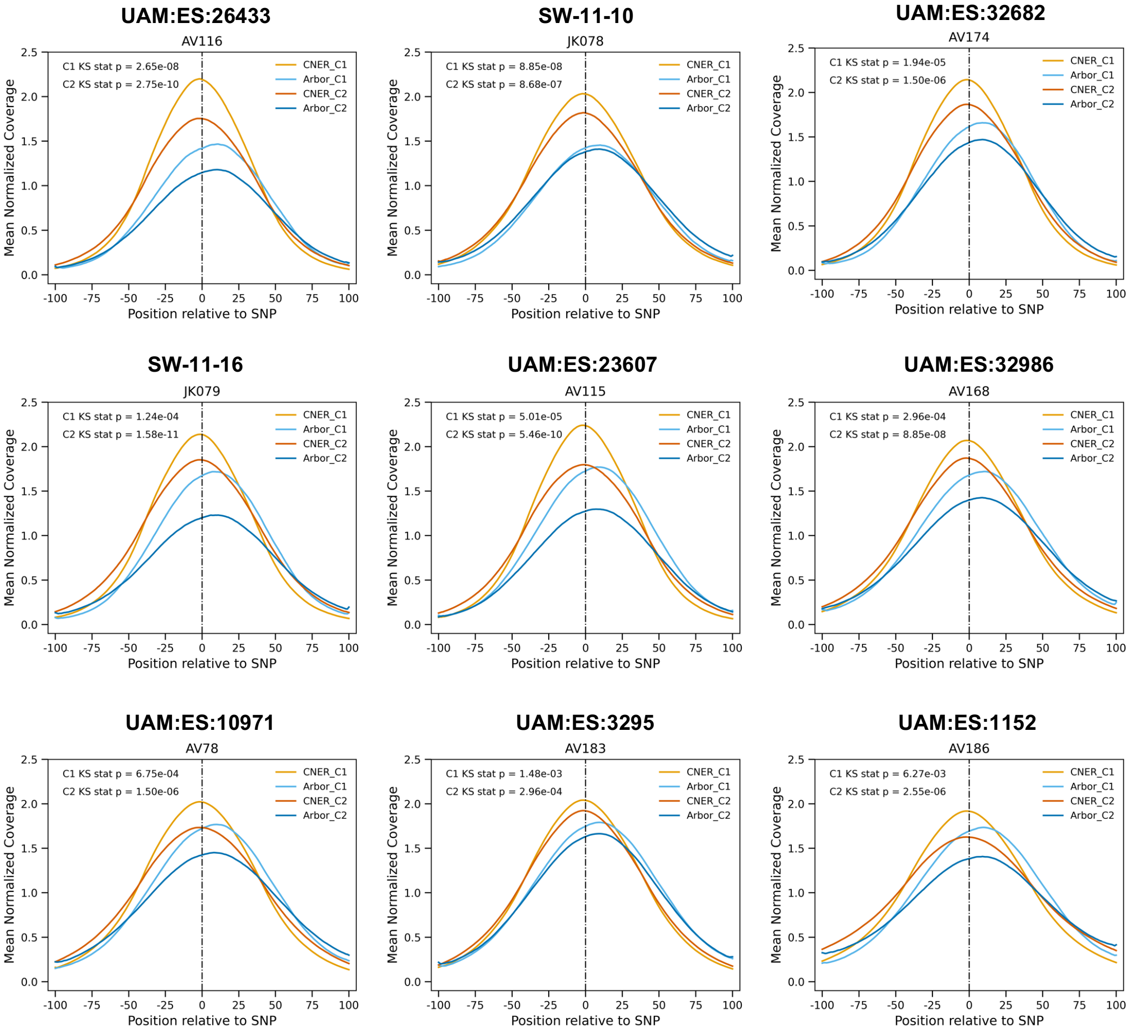

Figure S9: CNERs result in maximal coverage depth at SNP sites. Samples were individually captured by following the Arbor myBaits protocol in experiment A (labeled as ‘C1’) and by following CNERs protocol in experiment B (labeled as ‘C2’). CNERs were designed with SNP site at the center of the target regions. Plotting the coverage depth around the 200bp region (100bp on both sides) show that the read depth is maximum at the target SNP position and decreases on both sides regardless of the overall coverage and endogenous content of ancient samples (yellow lines). Coverage for Arbor myBaits (cyan lines) is shifted to right of the SNP site due to tilled probe design.

Figure S10: Genotype concordance between experiments and probes.

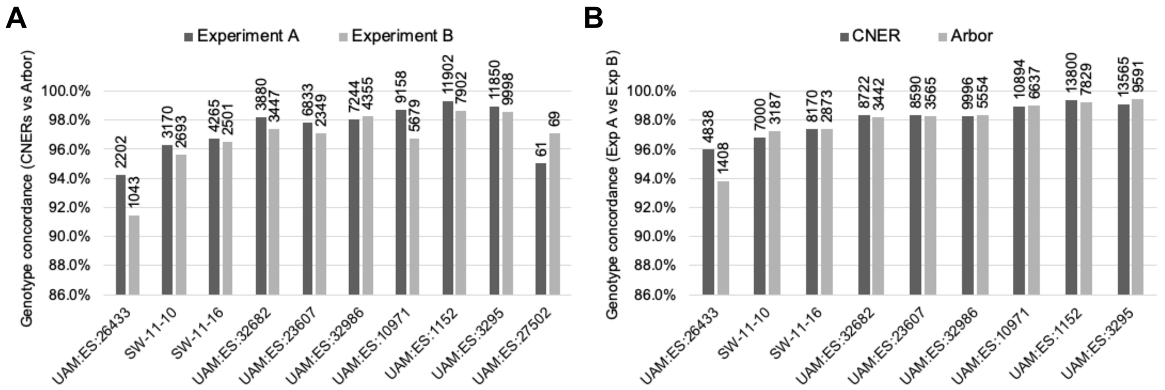

Figure S10: Genotype concordance between experiments and probes. (A) Genotype concordance (y-axis) between CNERs and Arbor myBaits is individually compared for experiment A (dark grey) and B (light grey) for ancient horse samples. **(B)** Genotype concordance (y-axis) between experiment A versus B is compared for CNERs captures (dark grey) and Arbor myBaits captures (light grey). Numbers above the bars indicate the number of SNPs genotypes between the two probes.

Figure S11: MapDamage plots showing Cytosine deamination modification at the ends of aDNA.

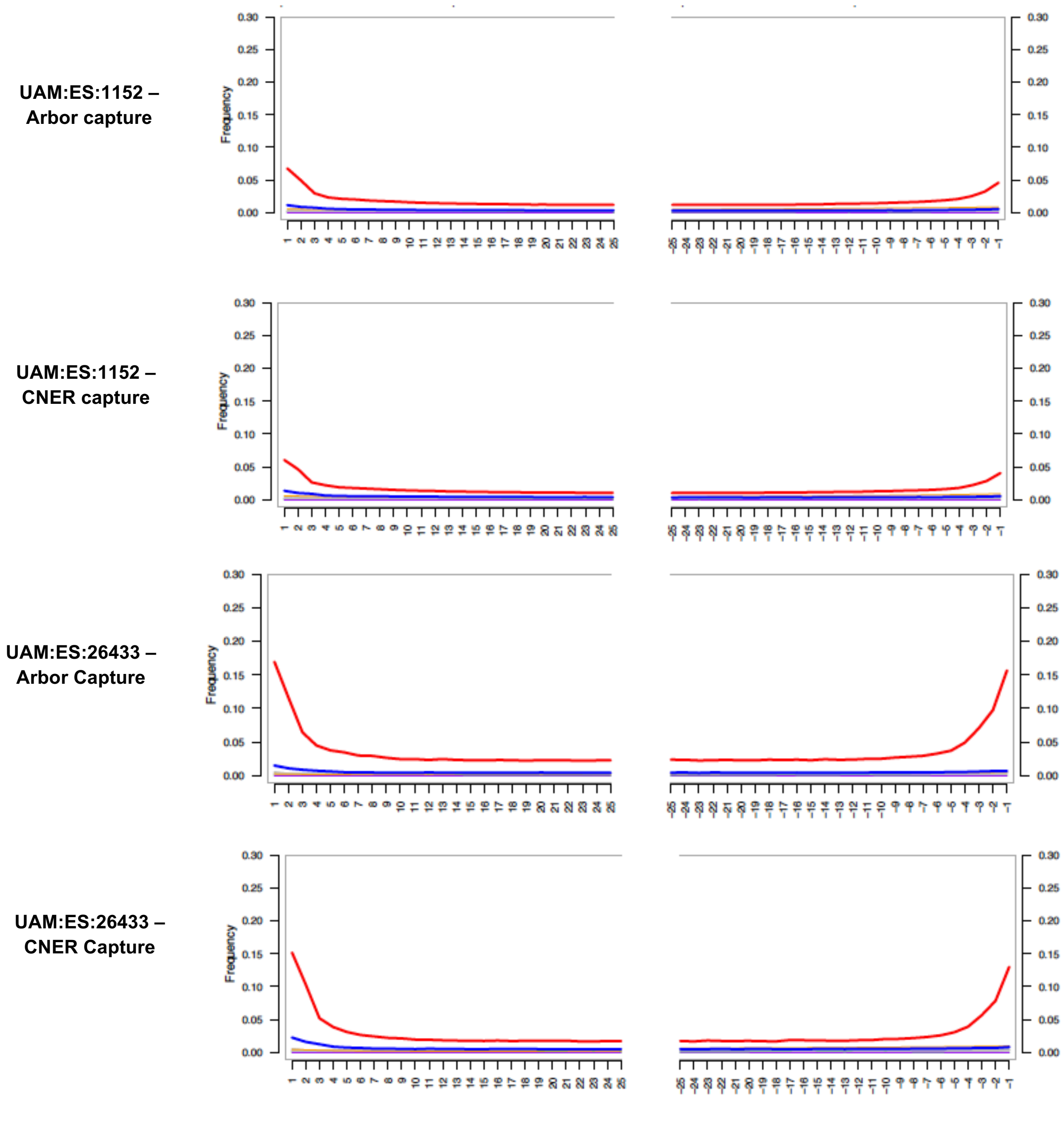

Figure S11: MapDamage plots showing Cytosine deamination modification at the ends of aDNA. Frequency of base substitutions from the end to 25bp internal to aDNA fragments are plotted using mapDamage program. The redlines show the C – to – T modifications which are elevated up to 6bp in both 5'and 3'ends. We plotted the mapDamage for two ancient horse samples as examples which show that there is no difference in the C-to-T modifications between CNERs and Arbor myBaits captured aDNA molecules from same horses.

Figure S12: CNERs enrich aDNA fragments with SNPs at the center.

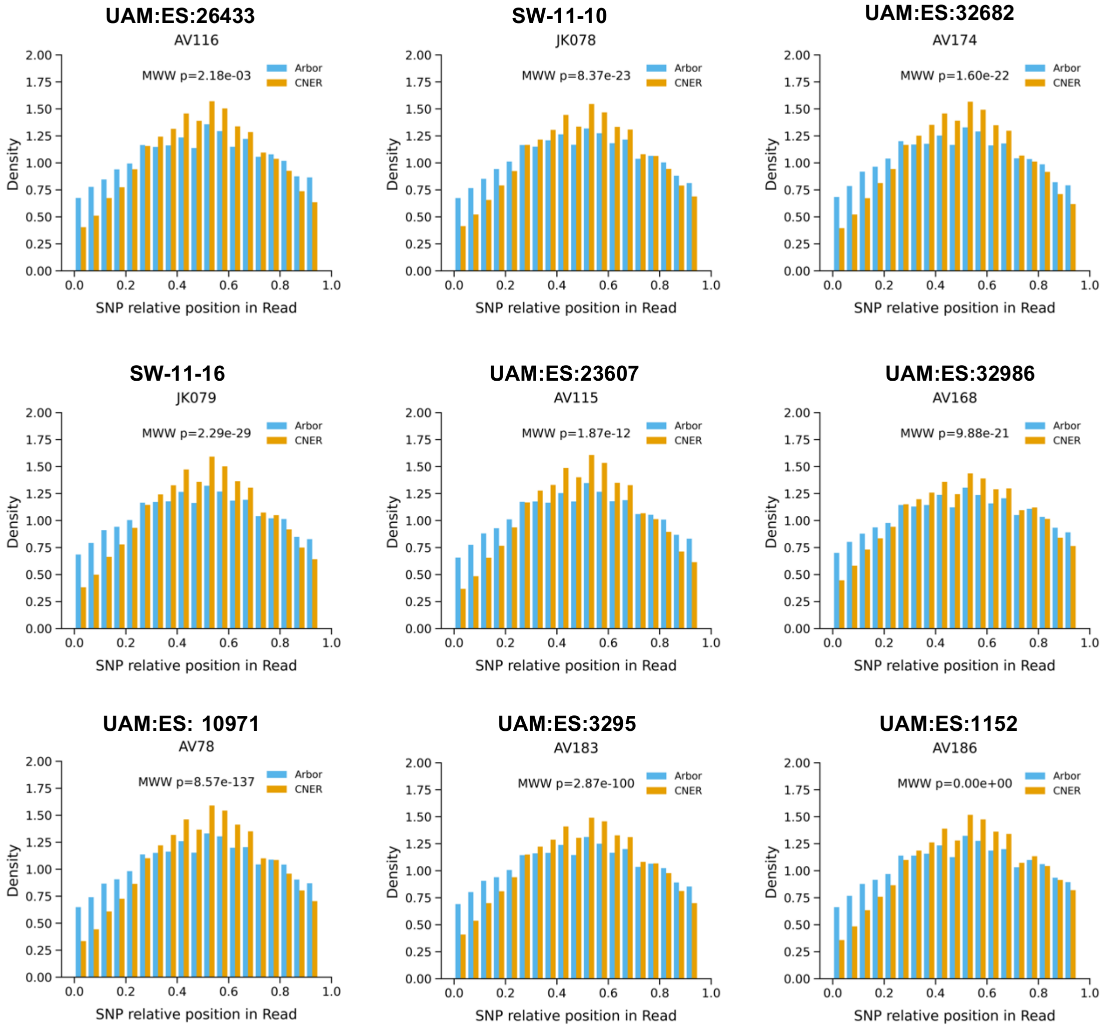

Figure S12: CNERs enrich aDNA fragments with SNPs at the center. Histogram of SNP position relative to the insert fragment length. x-axis shows the SNP position as a fraction of the read fragment length where 0 denotes the 5' end and 1 denotes the 3' end of the aDNA molecule. CNERs enrich aDNA fragments with SNPs at the center leading to the peak in the histogram at around 0.5. Whereas Arbor myBaits enrich aDNA fragments where SNP occurs almost anywhere in the molecules. Arbor myBaits significantly enrich more SNPs at the ends of the aDNA molecules than CNERs captures, p-value denotes the Mann-Whitney Wilcox U-test.

Figure S13: Admixture analysis for Arbor myBaits captured data.

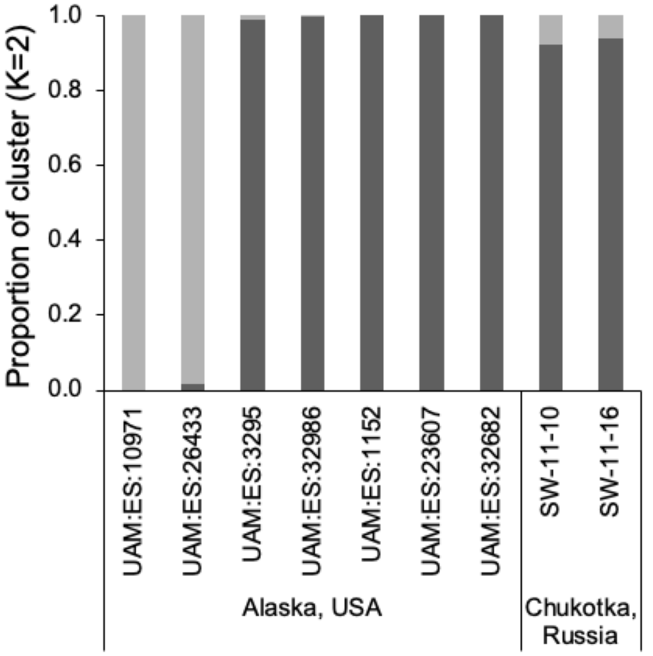

Figure S13: Admixture analysis with K=2 separated the ancient horses into two lineages regardless of their geographic location for Arbor myBaits captured data similar to CNERs data.
